# Design, microwave synthesis, and molecular docking studies of catalpol crotonates as potential neuroprotective agent of diabetic encephalopathy

**DOI:** 10.1038/s41598-020-77399-y

**Published:** 2020-11-23

**Authors:** Shuanglin Liu, Xiaodong Cheng, XiaoFei Li, Yuanfang Kong, Shiqing Jiang, Chunhong Dong, Guoqing Wang

**Affiliations:** 1 Henan University of Chinese Medicine, Zhengzhou, 450046 Henan China; 2 Department of Applied Chemistry, Zhengzhou University of Light Industry, Zhengzhou, 450002 Henan China

**Keywords:** Drug discovery, Chemistry

## Abstract

Catalpol has gained increasing attention for its potential contributions in controlling glycolipid metabolism and diabetic complications, which makes used as a very promising scaffold for seeking new anti-diabetic drug candidates. Acylation derivatives of catalpol crotonate (CCs) were designed as drug ligands of glutathione peroxidase (GSH-Px) based on molecular docking (MD) using Surfex-Docking method. Catalpol hexacrotonate (CC-6) was synthesized using microwave assisted method and characterized by FT-IR, NMR, HPLC and HRMS. The MD results indicate that with the increasing of esterification degree of hydroxyl, the C log *P* of CCs increased significantly, and the calculated total scores (Total_score) of CCs are all higher than that of catalpol. It shows that CCs maybe served as potential lead compounds for neuroprotective agents. It was found that the maximum Total_score of isomers in one group CCs is often not that the molecule with minimum energy. MD calculations show that there are five hydrogen bonds formed between CC-6 and the surrounding amino acid residues. Molecular dynamics simulation results show that the binding of CC-6 with GSH-Px is stable. CC-6 was screened for SH-SY5Y cells viability by MTT (3-(4, 5-dimethylthiazolyl-2)-2, 5-diphenyltetrazolium bromide) assay, the result indicates CC-6 can effectively reverse SZT induced cells apoptosis with dose-dependent manner, which can indirectly show that CC-6 is a potential neuroprotective agent.

## Introduction

**Figure 1:**
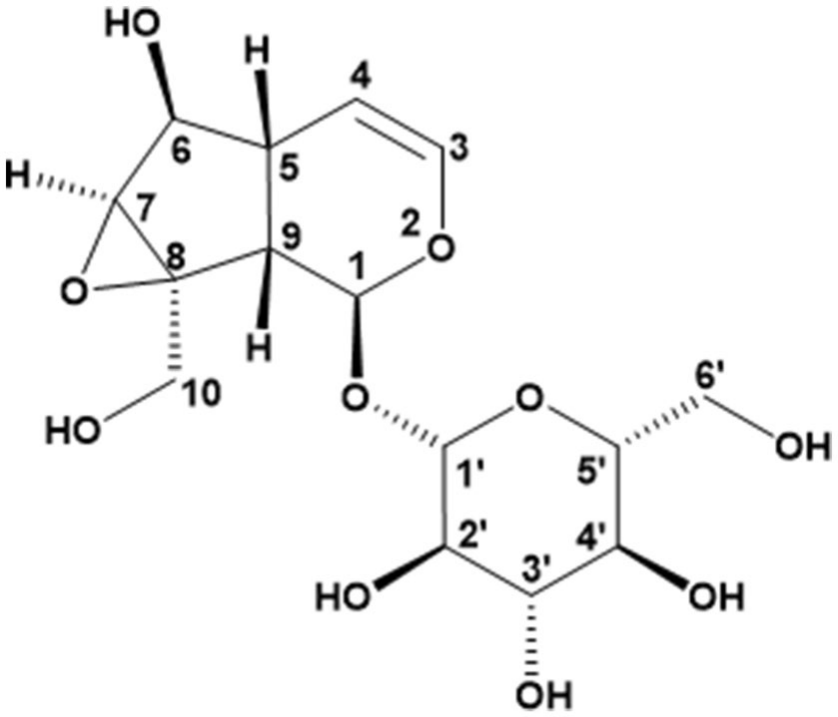
Structure of catalpol.

Catalpol is a kind of iridoid glycoside, which is widely found in several families of Plantaginaceae^1^, Lamiaceae and Bignoniaceae^2,3^. It is also the main active ingredient of *Rehmannia glutinosa Libosch.* which is one of the most commonly used herbs in traditional Chinese medicine for treatment of diabetes for more than 1000 years^4,5^. Modern pharmacological studies show that catalpol plays an important role in controlling glucose and lipid metabolism and diabetes complications^6,7^. Yang et al.^8^ revealed that catalpol had significant protective effect on morphological destruction and apoptosis induced by high glucose of the SH-SY5Y cell. Catalpol (Fig. 1), C_15_H_22_O_10_, is a polar and hydrophilic molecule with the glycoside bond sensitive to acid^9^. Catalpol was reported for its variety pharmacological activities, such as anti-oxidation, anti-inflammatory, antiapoptosis, antidiabetic and other neuroprotective properties^10–15^. It also plays a key role in the nerve protection of alzheimer's and Parkinson's disease both in vitro and in vivo models^10–12,15^.

However, catalpol is rarely reported to be used as a separate drug because of its blood–brain barrier caused by strong hydrophilicity and fast metabolism in vivo^16^. It is necessary to improve its lipophilicity and increase its utilization in vivo. It is known that esterification is a common method used for improving octanol–water partition coefficient (log *P*), membrane permeability, drug targeting and pharmacokinetics of hydrophilic compounds. Our preliminary studies found that catalpol propionates are better binding ligands with glutathione peroxidase (GSH-Px) than catalpol and show good anti-aging efficacy for its protection of SH-SY5Y cells^17^. There were a series of catalpol derivatives have been synthesized and reported by conventional chemical synthesis^17,18^, and these synthesis process were all time-consuming. Microwave assisted synthesis (MWAS) highlighted its great potential for synthesis of natural products and therapeutic agents with the advantage of time-saving^19^, but there is no report about MWAS applied for esterification of catalpol.

In this paper, the docking affinity (Total_score) between acylated derivatives of catalpol crotonic anhydride (CCs) and GSH-Px was calculated by using the Surfex-Docking method with the binding patterns visualized. The derivative catalpol hexacrotonate (CC-6) with the highest Total_score was synthesized using MWAS method and characterized by IR, NMR, UPLC-MS and HPLC. Molecular dynamics simulation was used to evaluated the stability of complex between CC-6 and GSP-Px. SH-SY5Y cells viability of CC-6 was screened for by MTT (3-(4, 5-dimethylthiazolyl-2)-2, 5-diphenyltetrazolium bromide) assay, and it was expected to obtain a potential neuroprotective agent of diabetic encephalopathy.

## Results and discussion

### Molecular docking study of CCs binding with GSH-Px

The structure of catalpol was shown as Fig. 1.

The position of hydroxyl groups in molecule structure of catalpol is marked as 6, 10, 2′, 3′, 4′, and 6′, respectively.The Total_score and octanol–water partition coefficient (C log *P*) of the CCs calculated based on Suflux method were shown in Table 1.

**Table 1:** Total_score of CCs docked with GSH-Px and their C log *P.*

Number^a^	Position^b^	Total_score	C log *P*	Position^b^	Total_score	C log *P*
0	/	4.27	− 4.38			
1	2′	4.33	− 3.36	6′	4.42	− 3.12
	3′	6.12	− 2.68	10 ^c^	4.57	− 2.46
	4′	5.92	− 2.68	6	5.14	− 2.46
2	4′, 6′	7.55	− 2.06	4′, 10	6.96	− 2.06
	2′, 6′	5.37	− 2.07	2′, 3′	7.88	− 1.63
	3′, 6′	5.51	− 1.42	3′, 6	4.53	− 0.77
	6′, 10 ^3^	5.15	− 1.20	3′, 10	6.07	− 1.57
	6′, 6	7.21	− 1.20	2′, 10	6.24	− 1.41
	3′, 4′	6.07	− 2.28	2′, 6	6.44	− 1.41
	2′, 4′	6.59	− 2.27	6, 10	5.16	− 0.55
	4′, 6	5.95	− 2.06			
3	4′, 6′, 10	7.12	− 0.14	2′, 4′, 6	8.06	− 0.35
	3′, 4′, 6′	8.95	− 0.36	2′, 3′, 4′	8.23	− 0.57
	2′, 4′, 6′	6.57	− 0.35	3′, 4′, 6	7.17	− 0.36
	4′, 6′, 6	7.80	− 0.14	3′, 4′, 10	8.51	− 0.36
	3′, 6′, 6	7.63	0.49	4′, 6, 10	7.38	− 0.14
	3′, 6′, 10	9.01	0.49	3′, 6, 10	5.74	1.15
	2′, 3′, 6′	6.21	− 0.37	2′, 3′, 6	7.55	0.28
	2′, 6′, 6	5.46	− 0.15	2′, 3′, 10	7.29	0.28
	2′, 6′, 10	9.70	− 0.15	2′, 6, 10	7.08	0.50
	2′, 4′, 10	6.92	− 0.35	6′, 6, 10 ^c^	4.55	0.71
4	2′, 6′, 6, 10	7.59	1.76	2′, 4′, 6, 10	8.78	1.56
	2′, 3′, 4′, 6′	7.67	1.34	3′, 4′, 6, 10	8.02	1.02
	3′, 4′, 6′, 6	8.31	1.55	2′, 3′, 6, 10	10.33	2.20
	3′, 4′, 6′, 10	8.95	1.55	2′, 4′, 6′, 6	8.10	1.56
	2′, 3′, 6′, 10	9.00	1.54	2′, 4′, 6′, 10	9.01	1.56
	2′, 3′, 6′, 6	7.73	1.54	4′, 6′, 6, 10 ^c^	9.01	1.77
	2′, 3′, 4′, 6	8.58	1.34	3′, 6′, 6, 10	8.42	2.41
	2′, 3′, 4′, 10	10.19	1.34			
5	2′, 3′, 6′, 6, 10	9.46	3.46	2′, 4′, 6′, 6, 10 ^c^	7.43	3.48
	2′, 3′, 4′, 6′, 6	7.79	3.26	2′, 3′, 4′, 6, 10	10.12	3.26
	2′, 3′, 4′, 6′, 10	7.73	3.26	3′, 4′, 6′, 6, 10	8.22	3.47
6	2′, 3′, 4′, 6′, 6, 10 ^c^	10.26	4.65			

^a^Number of crotonylated hydroxyl groups in CCs.

^b^Positions of crotonylated hydroxyl groups in CCs.

^c^The isomer with minimum molecular energy in CCs.

It can be seen from Table 1 that the Total_score and C log *P* of CCs increased significantly with the increase of the number of crotonate groups increased, and they were all higher than that of catalpol. Furthermore, it also can be seen that the Total_score and C log *P* reached their maximum values when hydroxyl groups in catalpol were all crotonylated. These results indicate that the crotonylation of hydroxy in catalpol maybe is an effective method to improve CCs’ log *P* and increase their druggability, and CC-6 is the best one among the 71 isomers. Meanwhile, it can be seen that the isomer with the highest Total_score in a group may not be the one with the lowest molecular structure energy, which means that the isomer with better pharmacological activity maybe not the one with more stable chemical structure.

**Figure 2:**
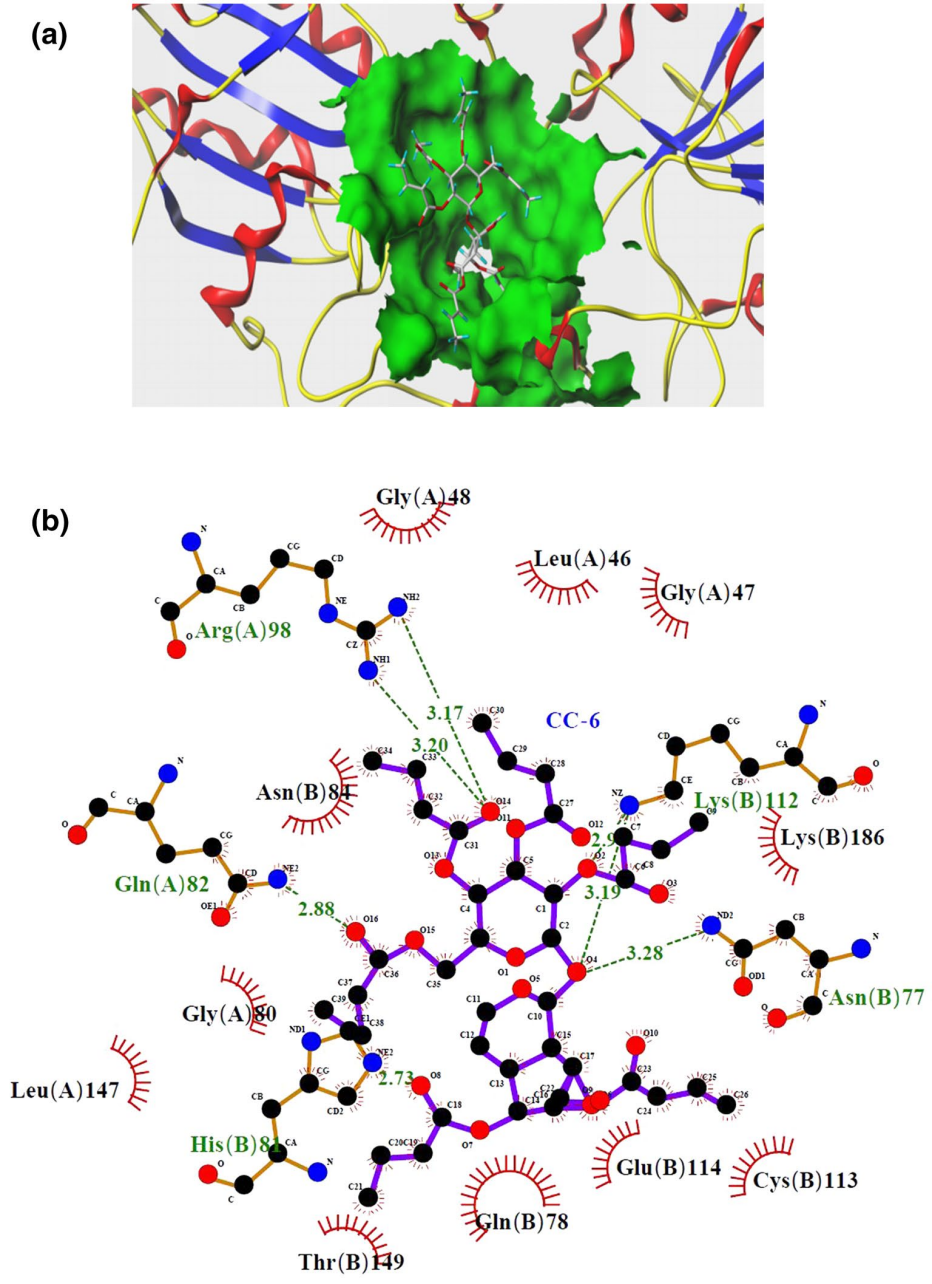
Docking diagram of CC-6 and GSH-Px molecule. (**a**) Docking result of CC-6 with GSH-Px; (**b**) Interaction of CC-6 with GSH-Px.

The interaction pattern between CC-6 and GSH-Px (PDB: 2f8a) amino acid residues was shown in Fig. 2.

In Fig. 2a, GSH-Px was represented by a ribbon model, CC-6 was represented by a capped stick, and the active site of the protein was represented as the green surface. In the Fig. 2b, the black ball represents the C atom, the red ball represents the O atom, the blue ball represents the N atom, the red eyelash character represents the hydrophobic interaction between CC-6 and GSH-Px, and the green dashed line is the hydrogen bond formed between the CC-6 and the amino acid residues around CC-6. It can be seen from Fig. 2b that the strong interaction between CC-6 and GSH-Px by the formation of hydrogen bondings and hydrophobic interactions with surrounding amino acid residues. Hydrophobic interactions form primarily via the carbon atoms in the ester part of CC-6 with nearby amino acid residues of Gly(A)48, Leu(A)46, Gly(A)47, Lys(B)186, Cys(B)113, Glu(B)114, Gln(B)78, Thr(B)149, Leu(A)147, Gly(A)80, Asn(B)84. The five hydrogen bonds between CC-6 and GSH-Px were formed by the interaction of oxygen atom, which in the carbonyl of ester group and in the glycosidic bond, with the nearby amino acid residues. The oxygen atom of glycosidic bond in CC-6 forms two hydrogen bonds with Asn(B)77 and Lys(B)112, respectively, the oxygen atom in ester carbonyl group at the 4′ position form two hydrogen bonds with Arg(A)98, the oxygen atom in ester carbonyl group at the 6′ position forms a hydrogen bondwith Gln(A)82.

### Molecular dynamics simulation of CC-6 with GSH-Px

**Figure 3:**
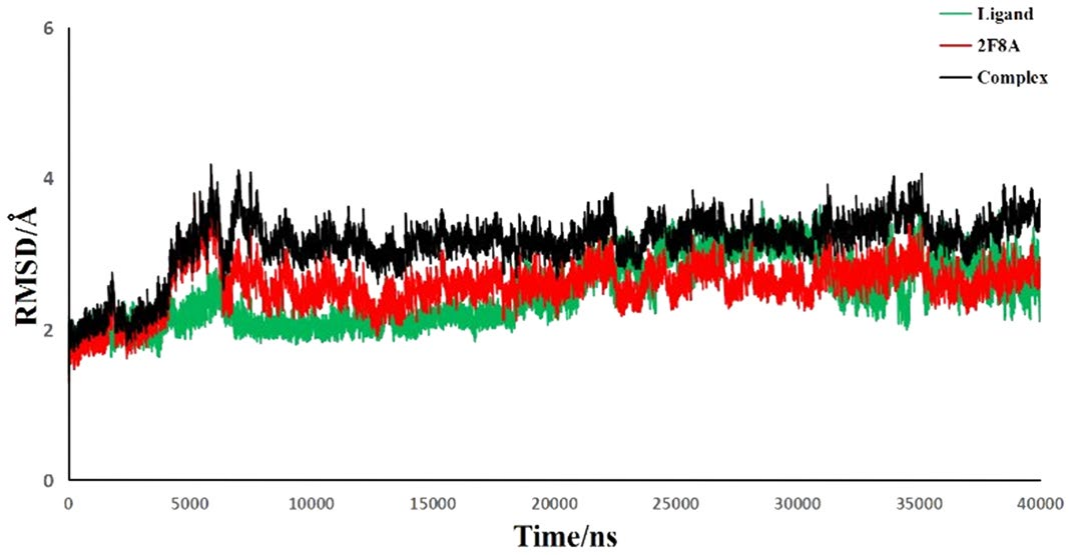
RMSD analysis of 40 ns molecular dynamic track of the system.

In order to predict the interaction stability of complexes between CC-6 and GSH-Px, the AMBER PACKAGE^20^ was used to conduct further dynamic simulation and systematically predict its macroscopic physical properties. Conformation of the system dynamics equilibrium stage were taken as the reference conformation, the root-mean square deviation (RMSD) changes of the GSH-Px skeleton atoms (CA, C, N) and the non-hydrogen atoms of the CC-6 in 40 ns were calculated and shown in Fig. 3.

It can be seen from Fig. 3 that the complexes of CC-6 and GSH-Px basically remained stable after 5000 ps, and the RMDS of the complex remained stable at about 3000 ps. These results indicate that the complexes of CC-6 and GSH-Px is stabl.

### Chemistry

For purpose of tracking the reaction process more accurately, ESI-HRMS was used for qualitative and semi-quantitative analysis of the products during the process of esterification of catalpol^17,21^. According to the result of ESI-HRMS analysis, it was found that the hydroxyl groups in catalpol were replaced one by one. Furthermore, with the reaction time prolonged, the amount of crotonic anhydride input more, and the reaction temperature increased, it was found that the darker of the color of the reaction mixture becomes. In order to obtain the optimal conditions of the MWAS process, orthogonal experimental design was performed.

#### Preparation and structure confirmation of CCs

**Scheme 1:**
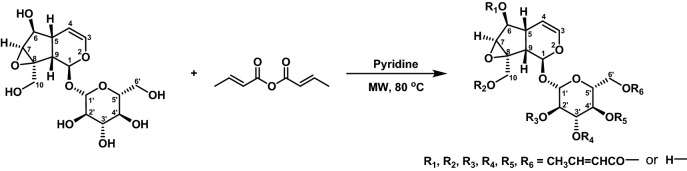
MWAS synthetic route of CCs.

The MWAS procedure used in this work was shown as Scheme 1. In this scheme, pyridine is not only acts as a solvent, but also acts as an acid binding agent. The molar ratio of reactants, amount of catalyst, reaction temperature and reaction time are the main factors affect the MWAS process. The optimal conditions for the orthogonal experimental design were as following as the molar ratio crotonicanhydride: catalpol = 18:1, the reaction temperature 80 °C, the power of reaction microwave 900 W, and the reaction time 8 h.

It was found that the relative content of target product in MWAS synthesis process was higher than that of the conventional method^17^, and the reaction time can be shortened by two thirds. CC-6 was purified by silica gel column chromatography, solid phase extraction column chromatography (SPE) and preparation-HPLC. It was found that preparation-HPLC was the best method for purification of CC-6.

#### Characterization of CC-6

**Figure 4:**
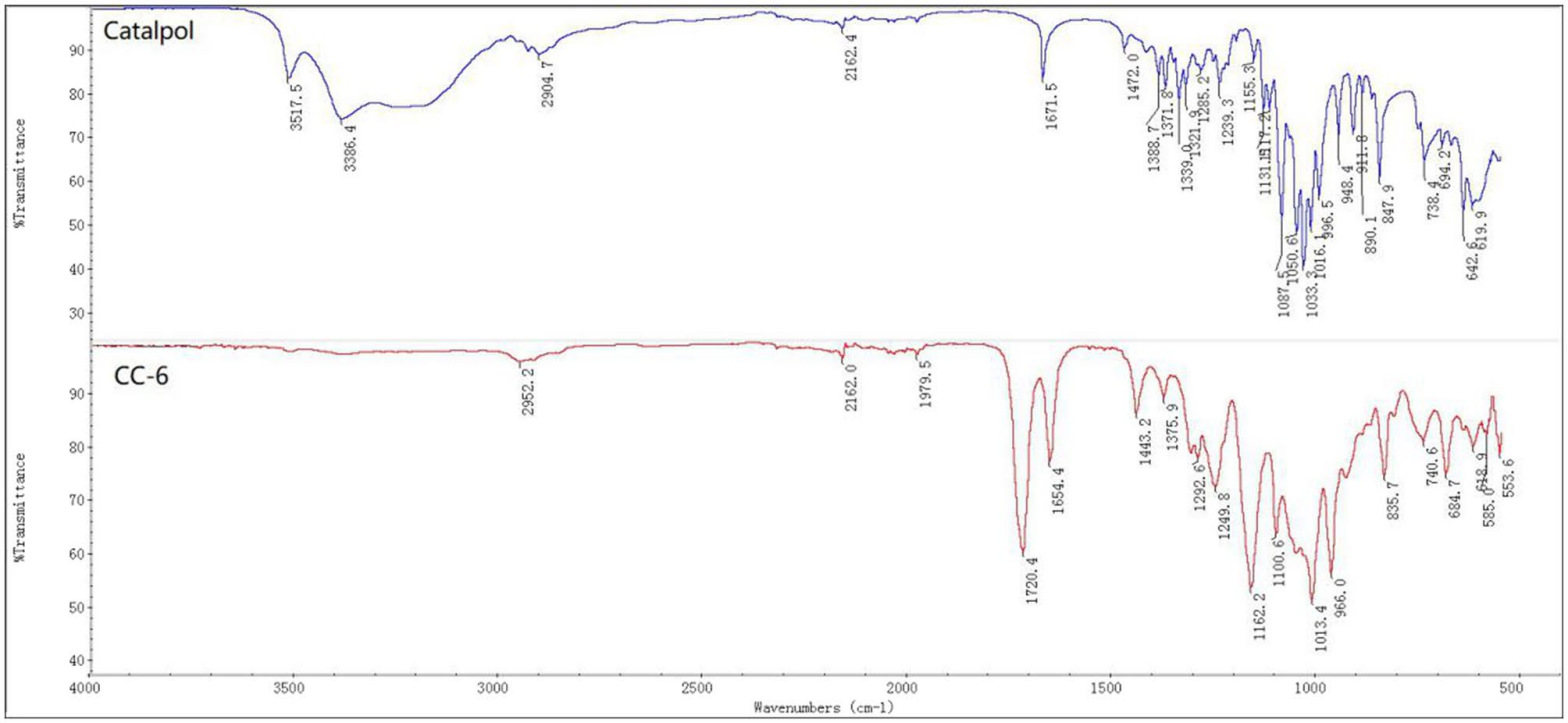
FT-IR spectra of catalpol (**a**) and CC-6 (**b**).

The chemical structure of CC-6 was confirmed by FT-IR, NMR, and UPLC-HRMS. The FT-IR spectra of catalpol and CC-6 were shown in Fig. 4a,b. It was found by comparing these two IR spectra that the typical strong absorption peak of the hydroxyl group at 3386.4 cm^−1^ can be seen in Fig. 4 (Catalpol), however it disappeared in Fig. 4 (CC-6), while the strong absorptive peak of ester carbonyl at 1720.4 cm^−1^ appeared . These preliminary result show that the six hydroxyl groups of the catalpol were all esterified with crotonoic anhydride.

**Figure 5:**
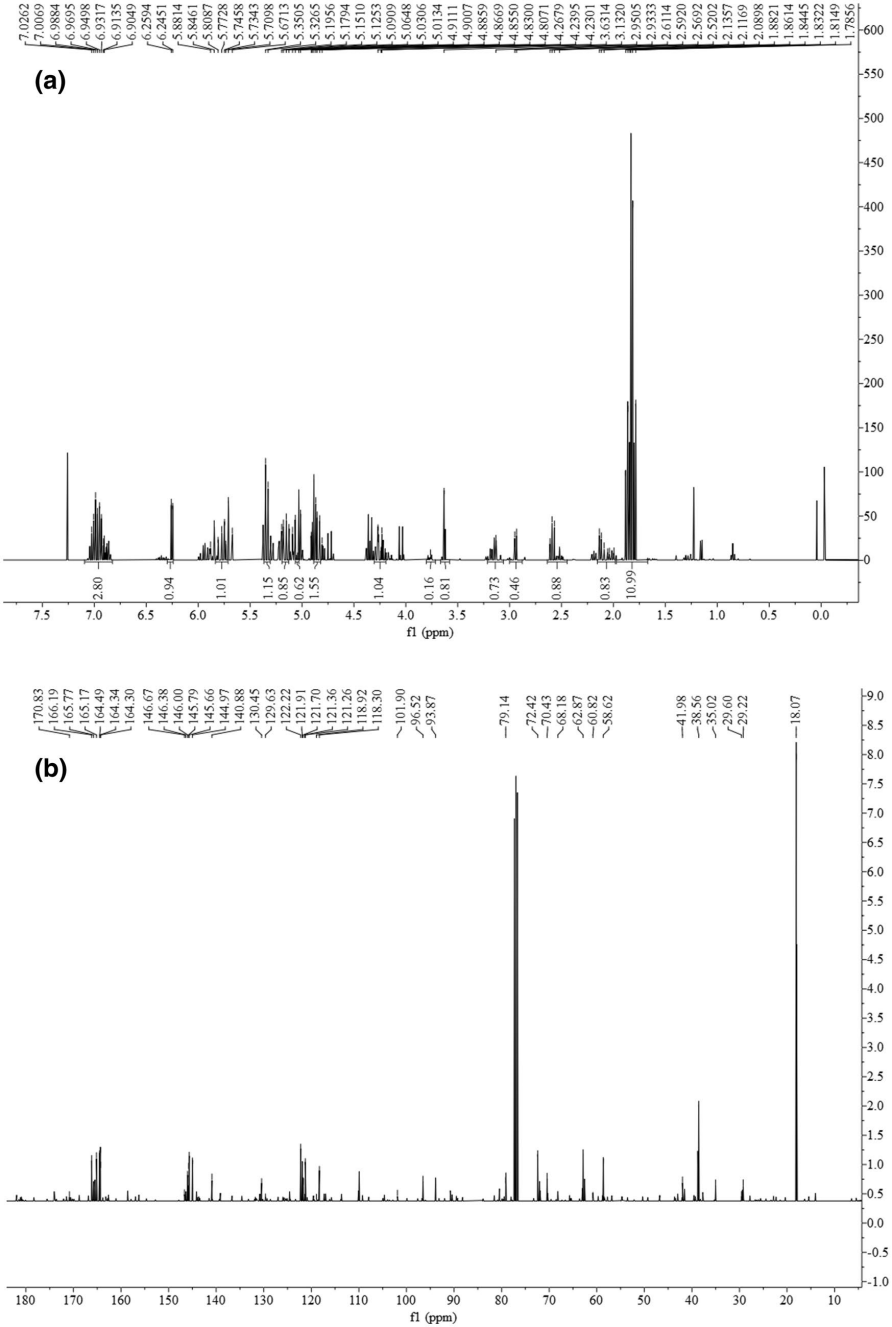
NMR spectra of CC-6. (**a**) ^1^H NMR spectrum of CC-6; (**b**) ^13^C NMR spectrum of CC-6.

The ^1^H NMR (400 MHz, CdCl_3_) and ^13^C NMR (100 MHz, CDCl_3_) of CC-6 were shown as Fig. 5a,b, respectively. In Fig. 5a, δ 7.03–6.90 (m, 6H, =CH–), 6.26–6.25 (d, *J* = 5.72 Hz, 1H), 5.88–5.67 (m, 3H), 5.35–5.33 (d, *J* = 9.60 Hz, 1H), 5.20–5.09 (m, 2H), 5.03–5.01 (m, 1H), 4.91–4.81(m, 2H), 4.27–4.23 (m, 2H), 3.76 (s, 1H), 3.63 (s, 1H), 3.13 (m, 2H), 2.95–2.93 (d, *J* = 6.88 Hz, 1H), 2.61–2.52 (m, 3H), 2.14–2.00 (m, 2H), 1.88–1.79 (m, 18H, CH_3_). In Fig. 5b, δ 170.83, 166.19, 165.77, 165.17, 164.49, 164.34, 164.30, 146.67, 146.38, 146.00, 145.79, 145.66, 144.97, 140.88, 130.45, 129.63, 122.22, 121.91, 121.70, 121.36, 121.26, 118.92, 118.30, 101.90, 96.52, 93.87, 79.14, 72.42, 70.43, 68.18, 62.87, 60.82, 58.62, 41.98, 38.56, 35.02, 29.60, 29.22, 18.07.

**Figure 6:**
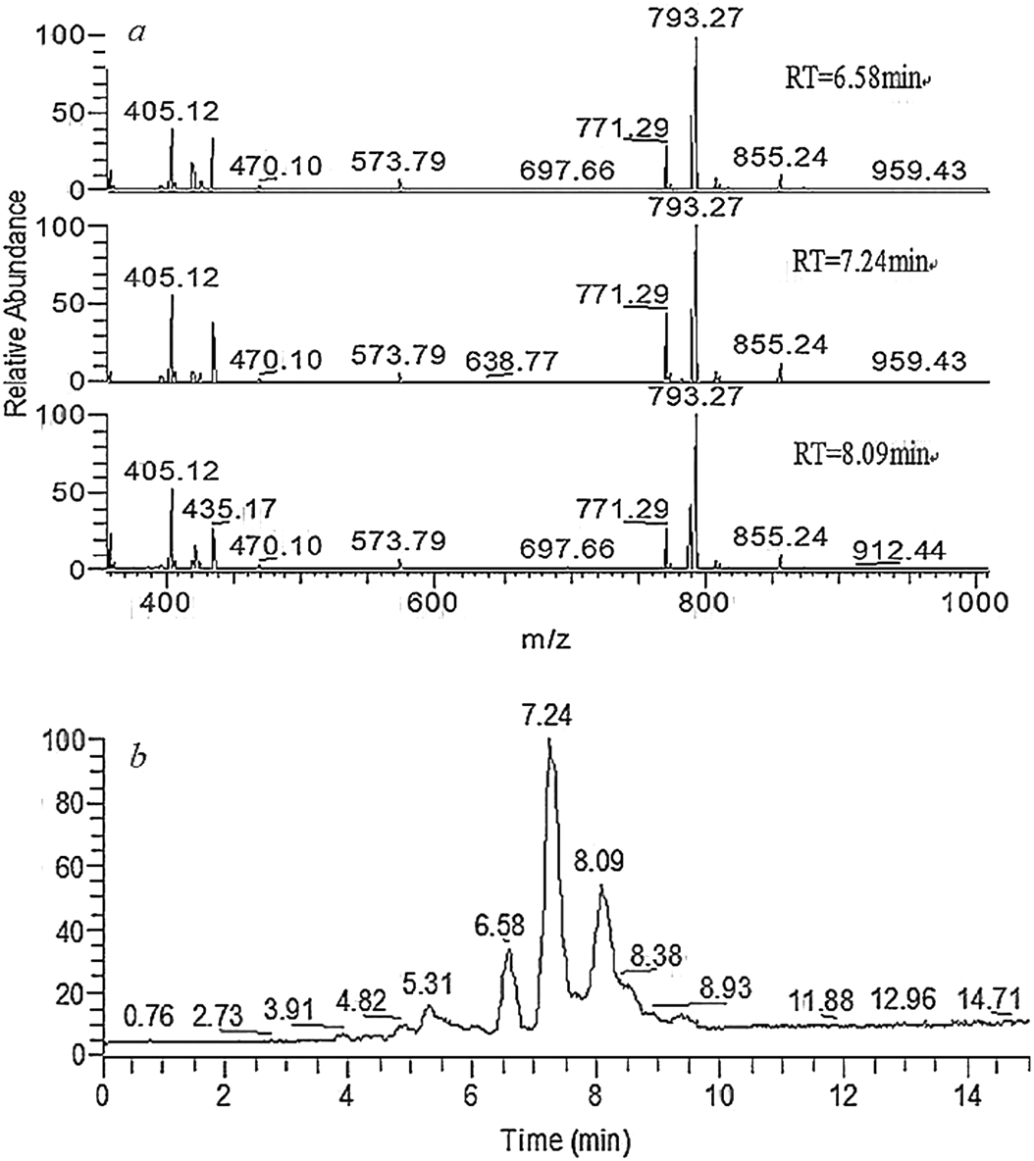
HRMS spectra and UPLC diagram on positive ion flow of CC-6.

The UPLC-HRMS of synthesized CC-6 under positive ion mode were shown as Fig. 6. The molecular formula of CC-6 is C_39_H_46_O_16_ with molecular weight 770.27, and two main ESI peaks (m/z) [M + H]^+^ 771.29, [M + Na]^+^ 793.27 at three retention times (min) 6.58, 7.24, and 8.09, respectively, are almost the same, this indicates that the six hydroxy groups of catalpol are all be crotonylated and formed CC-6. However, the retention behavior of CC-6 indicates that it is not composed of a single compound, but a mixed components with same molecular weight.

### Preliminary evaluation of neuroprotective effect

**Figure 7:**
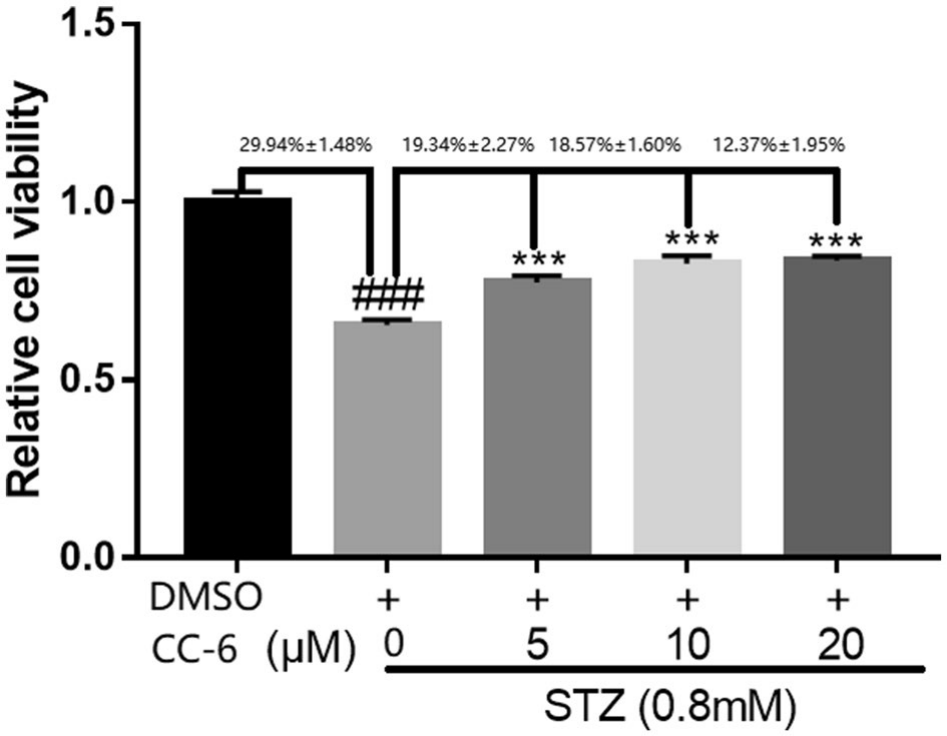
MTT assay for SH-SY5Y cells viability of CC-6.

The neuroprotective effect of CC-6 were detected by MTT assay six times at each condition, and the MTT assay results were shown as Fig. 7. In these assays, streptozotocin (STZ) were used to induced SH-SY5Y cell damage in vitro models, which has been widely used to induce glucose metabolism, neuronal apoptosis and tauopathy through oxidative damage. As demonstrated in Fig. 7, 0.8 mM STZ treatment induced a 29.94% ± 1.48% decrease in cell viability for SH-SY5Y, while co-incubation with 5, 10, 20 μM CC-6 effectively reversed STZ-induced reduction of cell viability 19.34% ± 2.27%, 18.57% ± 1.60%. and 12.37% ± 1.95%, with the confidence level 95%, respectively. These indicate that compound CC-6 has protective effects in neurons, and it is dose dependent. Therefore, CC-6 is a potential drug related to encephalopathy.

## Conclusion

CC-6 was synthesized using MWAS method with the advantage of time-saving and higher relative yield compared to that the conventional method. Molecular docking and dynamics simulations show that the complex formed by the combination of CC-6 and GSH-Px has good stability. The MTT assay results indicate that CC-6 has protective effects in neurons with dose-dependent manner, and it is a potential drug of diabetic encephalopathy.

## Experimental protocol

### Materials and apparatus

All of the reagents and solvents used were purchased from Aladdin Reagent (Shangha, China) Co., LTD without further purification. Microwave synthesizer was purchased from Kappler (Zhengzhou, China) Co., LTD. IR spectra were measured on KBr pellets with a NICOLET IS50 FT-IR spectrometer. NMR spectra were measured with BRUKER AV500MHz spectrometer. WATERS ACQUITY I CLASS & Exactive Obitrap high resolution mass spectrometer (UPLC-HRMS), ACQUITY UPLC BEH C18 (2.1 × 50 mm, 1.7 μm) column and PDA detector, ESI ionization source, automatic sampler, mobile phase ratio acetonitrile: 0.1% aqueous formate solution (V:V) = 90:10, flow rate 200 μL/min, capillary temperature 250 °C, capillary tube voltage 60 V, tube voltage 120 V, injection volume 1 μL, auxiliary gas 10 L/min, sheath gas 40 L/min, skimmer voltage 22 V, sketch time 15 min, scan range/(m/z) 360–800, electronic transmission tube temperature 275 °C, scanning mode positive ions scanning mode. MTT were evaluated with SpectraMax i3x Molecular Devices.

### Structure design

It was reported that GSH-Px has neuroprotective activity^23^, so the designed compounds binding with GSH-Px were selected for predict their neuroprotective activities. According to the consistency scoring function of CCs molecular docking with GSH-Px, their neuroprotective activity was predicted. Tripos force field was utilized for energy minimization, and followed by protomol generation^24^. The protomol was created by extracting the original ligand (PDB ID: 2f8a). All crystal water and small molecular ligands in protein crystals were removed. Hydrogen atoms with essential H-bond orientation and charge were added. In Multi-Channel Surface mode, several active pockets were generated and one of the best docking active pockets was selected, and the relevant side chains were repaired. The binding mode of the CCs with GSH-Px was calculated by adopting an empirical scoring function and a patented searching engine. The threshold is set to 0.50, and the expansion coefficient is set to 1 to form a prototype molecule for docking in high-precision mode. The original ligand in the active pocket as a reference was used to carry out the docking operation between the receptor and the ligand sets. Subsequently, a series of isomers of CCs were docked at the virtual active site via Geom X method by considering every ligand^25^. The binding affnity of the ligands is predicted in terms of Total_score which is expressed as − log10 *K*_d_, where *K*_d_ is binding constant.

### Molecular dynamics simulation

In order to further verify the rationality and stability of the docking results of CC-6 with GSH-Px, molecular dynamic simulation was conducted to predict their macroscopic physical properties using Amber package^20^. After hydrogenation of the whole system, ff14SB force field generation protein dynamics simulation were set through Leap module, and the small molecules is generated via the GAFF force field. The addition of gegenion sodium ions makes the system electrically neutral. Place the complex in a periodic hexahedral water box, the water molecules in the solvent were TIP3P with an intercept of 10 Å. During the dynamic simulation process, the Sander module is used to minimize the energy of the whole system in three steps. Firstly, 4000 steps of energy optimization in which the steepest descent method was used in the first 2000 steps, and then the conjugate gradient method was used in the next 2000 steps. Secondly, 5000 steps energy optimization in which steepest descent method was used in the first 2500 steps, then the conjugate gradient method was used in the next 2500 steps and limit the position of heavy atoms in the protein. Thirdly, 10,000 steps energy optimization: no binding force, unrestricted. The first 5000 steps steepest descent method, then another 5000 steps using conjugate gradient method. At last, the system temperature was gradually increased from 0 to 300 K within 0 ~ 50 ps, and the constant temperature was kept at 50 ~ 60 ps for 300 K in the NVT system, 40 ns dynamic simulation was performed for the complex system, and the simulation time step was set as 2 fs.

### Procedure for synthesis of CCs, characterization and purification of CC-6

The synthesis process of CCs was shown as Scheme 1, in which R1, R2, R3, R4, R5, R6 =CH_3_CH=CHCO– or H–, and the number of all the possible isomers is 71. 100.0 mg (0.276 mmol) catalpol was accurately weighed and put into micro reactor, then it was dissolved with 10 mL pyridine, 766 μL (4.968 mmol) crotonicanhydride was added to the reaction mixture slowly with stirring. Pyridine was used as both solvent and acid-binding agent. The resulting mixture was stirred and reacted at 70 °C assisted with microwave radiation at microwave output frequency 2450 MHz and output power 900 W. ESI-HRMS was used to monitor the synthesis process, and the reaction was terminated when the peak of catalpol was disappeared. The reaction was completed after 8 h, and then removal of pyridine by vacuum concentration using rotary evaporator. The residue concentrated of the reaction was extracted with 20 mL × 3 CHCl_3_, and then the organic layer was washed with 20 mL × 3 of saturated NaHCO_3_ solution, and dried over with MgSO_4_ overnight. After filtration, the crude product of CC-6 (220 mg) was obtained. CC-6 was purified separately by silica gel column chromatography, SPE-LC and preparation-HPLC. The purity of CC-6 of the purification procedure, silica gel column chromatography eluent: CH_2_Cl_2_: CH_3_OH = 5:1 (V/V) ), SPE-LC eluent CH_3_CN: H_2_O = 7:3 (V/V), preparation-HPLC (WATERS XBRIDGE C18, 10 μm, 19 × 150 mm; PDA detector), are 82.28% , 88.41% , and 96.88%, respectively, Whlie the yields (weight) of CC-6 are 60.21% (159.1 mg), 51.32% (126. 2 mg), and 72.68% (163.1 mg), respectively. The preparation-HPLC conditions were set as PDA detection wavelength of 210 nm, mobile phase CH_3_OH:H_2_O = 80:20 (V/V), and the flow rate 7 mL/min.

### MTT assay

Viability of cultured cells was detected by MTT assay^22^. The SH-SY5Y cells were seeded overnight with a density of 105 cells/well in 100 μL medium. The cells were co-incubated with different concentrations of CC-6 (5, 10, 20 μM) and STZ (0.8 μM) for 24 h. Then, the medium was removed and added 0.5 mg/μL MTT. After incubation at 37 °C for 4 h, 100 μL dimethyl sulfoxide (DMSO) was added to each well, and the mixture oscillate slowly for 10 min on a shaker to fully dissolve the formazan crystals, followed by the absorbance was measured at 490 nm with a SPECTRA MI3X spectrophotometer (Molecular Devices, USA).

## Data Availability

The datasets used or analysed during the current study are available from the corresponding author on reasonable request.
